# From Nash Equilibria to Chain Recurrent Sets: An Algorithmic Solution Concept for Game Theory

**DOI:** 10.3390/e20100782

**Published:** 2018-10-12

**Authors:** Christos Papadimitriou, Georgios Piliouras

**Affiliations:** 1 Computer Science Department, Columbia University, New York, NY 10027, USA; 2 Engineering Systems and Design (ESD), Singapore University of Technology and Design, Singapore 487372, Singapore

**Keywords:** algorithmic game theory, replicator dynamics, invariant, Kullback–Leibler divergence

## Abstract

In 1950, Nash proposed a natural equilibrium solution concept for games hence called Nash equilibrium, and proved that all finite games have at least one. The proof is through a simple yet ingenious application of Brouwer’s (or, in another version Kakutani’s) fixed point theorem, the most sophisticated result in his era’s topology—in fact, recent algorithmic work has established that Nash equilibria are computationally equivalent to fixed points. In this paper, we propose a new class of universal non-equilibrium solution concepts arising from an important theorem in the topology of dynamical systems that was unavailable to Nash. This approach starts with both a game and a learning dynamics, defined over mixed strategies. The Nash equilibria are fixpoints of the dynamics, but the system behavior is captured by an object far more general than the Nash equilibrium that is known in dynamical systems theory as *chain recurrent set.* Informally, once we focus on this solution concept—this notion of “the outcome of the game”—every game behaves like a potential game with the dynamics converging to these states. In other words, unlike Nash equilibria, this solution concept is algorithmic in the sense that it has a constructive proof of existence. We characterize this solution for simple benchmark games under replicator dynamics, arguably the best known evolutionary dynamics in game theory. For (weighted) potential games, the new concept coincides with the fixpoints/equilibria of the dynamics. However, in (variants of) zero-sum games with fully mixed (i.e., interior) Nash equilibria, it covers the whole state space, as the dynamics satisfy specific information theoretic constants of motion. We discuss numerous novel computational, as well as structural, combinatorial questions raised by this chain recurrence conception of games.

## 1. Introduction

Game theory has enjoyed a close relationship with the topology of dynamical systems from its very beginnings. Nash’s 1950 brilliant proof of the universality of equilibria is based on fixed point theorems—the most sophisticated class of topological theorems of that time, stating that all continuous dynamics have a fixpoint. Recent work in algorithmic game theory has brought these two concepts—Nash equilibria and fixpoints—even closer by establishing that they are in a formal sense computationally equivalent [1].

The Nash equilibrium is the foremost solution concept in game theory—the standard notion of *outcome* of the game. However, natural dynamics do not always converge to a Nash equilibrium, and there are results suggesting that no dynamics can converge to the Nash equilibrium in every game [2,3]. It is therefore interesting to ask, *to which object do natural dynamics converge?*

One way to answer this question is suggested by the fundamental theorem of dynamical systems, the pinnacle of modern dynamical systems theory introduced in the seminal work of Conley [4] in 1978. The theorem states that, given an arbitrary initial condition, a dynamical system converges to a set of states with a natural recurrence property akin to periodicity, explained next.

Conley’s concept of recurrence—a subtle generalization of cycling and periodicity—is elegant in a way that should appeal to any student of computation, and especially cryptography. Imagine that Alice is trying to simulate the trajectory of a given system on a powerful computer. Every time she computes a single iteration of the dynamical process, there is a rounding error ϵ. Furthermore, imagine that inside the machine there is an infinitely powerful demon, Bob who, before the next computational step is taken, rounds the result in arbitrary fashion of his own choosing (but within distance ϵ of the actual outcome). If, no matter how high the accuracy of Alice’s computer is, Bob can always fool her into believing that the starting point is periodic, then this point is a chain recurrent point.

Naturally, periodic points are chain recurrent, since Bob does not have to intervene. Therefore, equilibria are also chain recurrent since they are trivially periodic. On the other hand, there exist chain recurrent points that are not periodic. Imagine for example a system that is defined as the product of two points moving at constant angular speed along circles of equal length, but where the ratio of the speed of the two points is an irrational number, e.g., $\sqrt{2}$. This system will, after some time, get arbitrarily close to its initial position and a slight bump of the state by Bob would convince Alice that the initial condition is periodic. Thus, chain recurrent points are the natural generalization of periodic points in a world where measurements may have arbitrarily high, but not infinite, accuracy. The surprising implication of the fundamental theorem of dynamical system is that this generalization is not just necessary when arguing about computational systems but also sufficient. It captures all possible limit behaviors of the system.

How does this connect back to game theory? We can apply the fundamental theorem of dynamical systems on the system (G,f) that emerges by coupling game *G* and learning dynamics *f*. (For most of this paper, we take *f* to be replicator dynamics [5], that is, the continuous-time version of Multiplicative Weights Update [6]). This defines a new solution concept, the set of chain recurrent points of the system, which we denote R(G,f) or simply R.

**Our results.***We characterize the set of chain recurrent points for replicator dynamics for two classic and well-studied classes of games, zero-sum games and potential games (as well as variants thereof).* For zero-sum games with fully mixed (i.e., interior) Nash equilibria, the set of chain recurrent points of replicator dynamics is equal to the whole state space (i.e., R is all (randomized) strategy profiles). For potential games, R is minimal coinciding with the set of equilibria/fixed points of the (replicator) dynamics. We discuss how our results are robust as they straightforwardly extend to natural variants of the above games, i.e., affine/network zero-sum games and weighted potential games. Thus, an interesting high level picture emerges. In games with strongly misaligned incentives, R tends to be large implying unpredictability of the dynamics, whereas in games with strongly aligned incentives R tends to be small implying that learning/adaptation is easier in collaborative environments.

**Zero-sum games.** In zero-sum games, we show that, if a fully mixed Nash equilibrium exists (like in Matching Pennies), then the set of chain recurrent states is the whole state space (Theorem 6). This seems to fly in the face of the conventional game theoretic wisdom that in a zero-sum game with a unique maxmin/Nash equilibrium, all natural, self-interested dynamics will “solve” the game by converging swiftly to the maxmin. It is well known that convergence is restored if one focuses not on behavior, but on the time-average of the players’ utilities, strategies [7]; however, time averages are a poor way to capture the actual system behavior.

More generally, in zero-sum games with fully mixed (interior) equilibria replicator dynamics exhibit constants of motion [5]. Combining this with the fact that replicator dynamics can be shown to preserve “volume”, this implies that almost all initial conditions are (Poincaré) recurrent. That is, almost all initial conditions return arbitrarily close to themselves. Rounding errors can cause the system to jump from one trajectory to a nearby one. No matter how small these jumps are, one can fully migrate from any point of the phase space to any other, rendering the system completely unpredictable in the presence of an arbitrarily small and infrequent perturbation.

**(Weighted) potential games.** On the contrary, for potential games, it is already well known that replicators (and many other dynamics) converge to equilibria (e.g., [5,8]). As the name suggests, natural dynamics for these games have a Lyapunov/potential function that strictly decreases as long as we are not at equilibrium. As we show, unsurprisingly, this remains true for the more expansive class of weighted potential games. One could naturally come to hypothesize that, in such systems, known in dynamical systems theory as *gradient-like*, the chain recurrent set must be equal to the set of equilibria. However, this is not the case as one can construct artificial counterexamples where the limit points of each trajectories have a unique equilibrium but the whole statespace is chain recurrent.

Here is a counterexample that is due to Conley [4]. Imagine a continuous dynamical system on the unit square, where all points on the boundary are equilibria and all other trajectories flow straight downwards. This is a gradient-like system since the height *y* is always strictly decreasing unless we are at an equilibrium. Nevertheless, by allowing hops of size ϵ, for any ϵ>0, we can return from every point to itself. Starting from any (x,y), we move downwards along the flow, and when we get close enough to the boundary, we hop to the point (x,0). Afterwards, we use these hops to traverse the boundary until we reach point (x,1), and then one last hop to (x,1−ϵ) places us on a trajectory that will revisit our starting point. Hence, once again, the whole state space is chain recurrent despite the fact that the system has a Lyapunov function. The situation is reminiscent of the game of snakes and ladders. The interior states correspond to snakes that move you downwards, whereas the boundary and the ϵ perturbations work as ladders that you can traverse upwards. Unlike the game of snakes and ladders, however, there is no ending state in this game and you can keep going in circles indefinitely.

In the case of (weighted) potential games, we show that such contrived counterexamples cannot arise (Theorem 8). The key component to this proof is to establish that the set of values of the potential function over all system equilibria is of zero measure. We prove this by using tools from analysis (Sard’s theorem). Thus, we establish that, as we decrease the size of allowable perturbations, eventually these perturbations do not suffice for any new recurrent states to emerge and thus the set of equilibria and chain recurrent points of replicator dynamics coincide.

## 2. Related Work

Studying dynamics in game theoretic settings is an area of research that is effectively as old as game theory itself. Nevertheless, already from its very beginnings with the work of Brown and Robinson [9,10] on dynamics in zero-sum games, the study of dynamics in game theory played a second fiddle to the main tune of Nash equilibria. The role of dynamics in game theory has predominantly been viewed as a way to provide validation and computational tools for equilibria.

At the same time, starting with Shapley [11], there has been an ever increasing trickle of counterexamples to the dominant Nash equilibrium paradigm that has been building up to a steady stream within the algorithmic game theory community ([12,13,14,15,16,17,18]). Several analogous results are well known within evolutionary game theory [5,19]. Replicator dynamics [20] is known to be a good model of stochastic learning behavior under frequent play and slow movement creating a formal bridge between these non-equilibrium results. These observations do not fit the current theoretical paradigm.

Recent intractability results in terms of computing equilibria [1,21] have provided an alternate, more formal tone to this growing discontent with the Nash solution concept, however, the key part is still missing. We need a general theory that fully encompasses all these special cases.

The definition of chain recurrent sets as well as a reference to the fundamental theorem of dynamical systems have been actually introduced in what is currently the definitive textbook reference of evolutionary game theory [19]; however, the treatment is rather cursory, limited to abridged definitions and references to the dynamical systems literature. No intuition is built and no specific examples are discussed.

One recent paper [3] is quite related to the issues examined here. In this work, Benaïm, Hofbauer, and Sorin establish stability properties for chain recurrence sets in general dynamical systems. Applications to game theory are discussed. It is shown that there exist games where chain recurrent sets for learning dynamics can be more inclusive than Nash. However, no explicit characterization for these sets is established and all fully mixed (i.e., interior) initial conditions actually converge to Nash. Connections between chain recurrence sets in potential games and equilibria are discussed without formal proofs. Our results provide complete characterizations of chain recurrent sets and reflect the realized behavior given generic initial states. We believe that chain recurrent sets is a central object of interest whose properties need to be carefully analyzed and we set up a series of analytical goals along these lines in the future work section.

**Follow-up work:** In follow-up work to our conference version paper [22], Mertikopoulos, Piliouras and Papadimitriou [18] showed how to generalize the proofs of recurrent behavior for replicator dynamics in (network) zero-sum games by Piliouras and Shamma [23] to more general dynamics, i.e., the class of continuous time variants of follow the regularized leader (FTRL) dynamics. Bailey and Piliouras [24] studied the discrete-time version of FTRL dynamics in (network) zero-sum games (including Multiplicative Weights Update) and showed how they diverge away from equilibrium and towards the boundary. Piliouras and Schulman [25] show how to prove periodicity for replicator dynamics in the cases of non-bilinear zero-sum games. Specifically, they consider games that arise from the competition between two teams of agents where all the members in the team have identical interests but where the two teams have opposing interests (play a constant sum game). Periodicity for replicator dynamics has also been established for triangle-network zero-sum games [26]. Recurrence arguments for zero-sum games can also be adapted in the case of dynamically evolving games [27]. FTRL dynamics can be adapted so that they converge in bilinear [28] (or even more general non-convex-concave [29]) saddle problems. Such algorithms can be used for training algorithms for generative adversarial neural networks (GANs). Finally, Balduzzi et al. show how to explicitly “correct” the cycles observed in dynamics of zero-sum games by leveraging connections to Hamiltonian dynamics and use these insights to design different algorithms for training GANs [30].

## 3. Preliminaries

### 3.1. Game Theory

We denote an *n*-agent game as $\left(n,\times_{i}S_{i},\times u_{i}\right)$. Each agent chooses a strategy $s_{i}$ from its set of available strategies $S_{i}$. Given a strategy profile $s=(s_{1},\cdots ,s_{n})$, the payoff to each agent *i* is defined via its utility function $u_{i}:\times_{i}S_{i}\to I\;R$. Every potential game has a potential function $\Phi :\times_{i}S_{i}\to I\;R$, such that at any strategy profile *s* and for each possible deviation of agent *i* from strategy $s_{i}$ to $s_{i}^{\prime }$: $\Phi \left(s_{i}^{\prime },s_{-i}\right)-\Phi \left(s_{i},s_{-i}\right)=u_{i}\left(s_{i}^{\prime },s_{-i}\right)-u_{i}\left(s_{i},s_{-i}\right)$. A weighted potential game has a potential function $\Phi :\times_{i}S_{i}\to I\;R$, such that at any strategy profile *s* and for each possible deviation of agent *i* from strategy $s_{i}$ to $s_{i}^{\prime }$: $\frac{\Phi \left(s_{i}^{\prime },s_{-i}\right)-\Phi \left(s_{i},s_{-i}\right)}{w_{i}}=u_{i}\left(s_{i}^{\prime },s_{-i}\right)-u_{i}\left(s_{i},s_{-i}\right)$ for some agent specific $w_{i}>0$. Wlog we can assume $w_{i}\geq 1$ for all agents *i*, by scaling Φ as needed. Naturally, the definitions of strategy and utility can be extended in the usual multilinear fashion to allow for randomized strategies. In that case, we usually overload notation in the following manner: if $x_{i}$ is a mixed strategy for each agent *i*, then we denote by $u_{i}\left(x\right)$ the expected utility of agent *i*, $E_{s∼x}\left[u_{i}\left(s\right)\right]$. We denote by $x_{is_{i}}$ the probability that agent *i* assigns to strategy $s_{i}\in S_{i}$ in (mixed) strategy profile *x*. To simplify notation, sometimes instead of $x_{is_{i}}$ we merely write $x_{s_{i}}$.

### 3.2. Replicator Dynamics

The replicator equation [31,32] is described by:

$$\frac{dp_{i}\left(t\right)}{dt}={\dot{p}}_{i}=p_{i}\left[u_{i}\left(p\right)-\hat{u}\left(p\right)\right],\;\hat{u}\left(p\right)=\sum_{i=1}^{n}p_{i}u_{i}\left(p\right),$$

where $p_{i}$ is the proportion of type *i* in the population, $p=(p_{1},\ldots ,p_{m})$ is the vector of the distribution of types in the population, $u_{i}\left(p\right)$ is the fitness of type *i*, and $\hat{u}\left(p\right)$ is the average population fitness. The state vector *p* can also be interpreted as a randomized strategy. Replicator dynamics enjoy connections to classic models of ecological growth (e.g., Lotka–Volterra equations [5]), as well as discrete time dynamics, e.g., Multiplicative Weights Update algorithm (MWU) [6,8,33].

**Remark** **1.** *In the context of game theory $p_{i}$ will be replaced with $x_{is_{i}}\left(t\right)$, i.e., the probability that agent i plays strategy $s_{i}\in S_{i}$ at time t.*

**Replicator dynamics as the “fluid limit” of MWU.** The connection to Multiplicative Weight Updates (MWU) [6] is of particular interest and hence it is worth reviewing briefly here. MWU is an online learning dynamics where the decision maker keeps updating a weight vector which can be thought informally as reflecting the agent’s confidence that the corresponding actions will perform well in the future. In every time period, the weights are updated multiplicatively (as the name suggests) $w_{s_{i}}^{t+1}\leftarrow w_{s_{i}}^{t}{\left(1+\epsilon \right)}^{u^{t}\left(s_{i}\right)}$. In the next time period, the agent chooses each action with probability proportional to its weight. As long as the learning rate ϵ is a small constant (or even better decreasing with time, e.g., at a rate $1/\sqrt{t}$), then it is well known that MWU has good performance guarantees in online (or even adversarial) environments, i.e., low regret [34]. When all agents in a game apply MWU with learning parameter ϵ (MWU(ϵ)), this defines a deterministic map from the space of weights (or after normalization from the space of probability distributions over actions) to the space of probability distributions over actions. If we define this map from the space of mixed strategy profiles to itself as f(ϵ), then the replicator vector field corresponds to the coefficient of the first order term in the Taylor expansion of *f* as a function of ϵ. In other words, the replicator vector field is equal to ${\frac{\partial f}{\partial \epsilon }|}_{\epsilon =0}$, a first order “smooth” approximation of the expected motion of the discrete time map MWU(ϵ). This argument was first exploited in [8] to study MWU dynamics in potential games.

Both replicator and MWU have strong connections to entropy. They can be interpreted as solutions to a softmax problem where the agent chooses the (mixed) strategy that maximizes his expected payoff (if each action was assigned value equal to its accumulated payoff) minus the (negative) entropy of the strategy. The (negative) entropy of the distribution is employed as a convex regularizer encouraging the algorithm to pick more “mixed” strategies. These connections are discussed in more detail in the following papers [18,35].

### 3.3. Topology of Dynamical Systems

In an effort to make our work as standalone as possible, we provide an introduction to some key concepts in topology of dynamical systems. The presentation follows along the lines of [36], the standard text in evolutionary game theory, which itself borrows material from the classic book by Bhatia and Szegö [37]. The exposition on chain recurrence follows from [38].

**Definition** **1.** *A flow on a topological space X is a continuous function ϕ:IR×X→X such that*

*(i)*  *ϕ(t,·):X→X is a homeomorphism for each t∈IR.*
*(ii)*  *ϕ(s+t,x)=ϕ(s,(ϕ(t,x))) for all s,t∈IR and all x∈X.*

The second property is known as the group property of the flows. The topological space *X* is called the phase (or state) space of the flow.

**Definition** **2.** *Let X be a set. A map (or discrete dynamical system) is a function f:X→X.*

Typically, we write $\phi^{t}\left(x\right)$ for ϕ(t,x) and denote a flow ϕ:IR×X→X by $\phi^{t}:X\to X$, where the group property appears as $\phi^{t+s}\left(x\right)=\phi^{s}\left(\phi^{t}\left(x\right)\right)$ for all x∈X and s,t∈IR. Sometimes, depending on context, we use the notation $\phi^{t}$ to also signify the map ϕ(t,·) for a fixed real number *t*. The map $\phi^{1}$ is useful to relate the behavior of a flow to the behavior of a map.

**Definition** **3.** *If $\phi^{t}$ is a flow on a topological space X, then the function $\phi^{1}$ defines the time-one map of $\phi^{t}$.*

Since our state space is compact and the replicator vector field is Lipschitz-continuous, we can present the unique solution of our ordinary differential equation by a flow ϕ:IR×X→X, where *X* denotes the set of all mixed strategy profiles. Fixing starting point x∈X defines a function of time which captures the trajectory (orbit, solution path) of the system with the given starting point. This corresponds to the graph of ϕ(·,x):IR→X, i.e., the set {(t,y):y=ϕ(t,x)forsomet∈IR}.

If the starting point *x* does not correspond to an equilibrium, then we wish to capture the asymptotic behavior of the system (informally the limit of ϕ(t,x) when *t* goes to infinity). Typically, however, such functions do not exhibit a unique limit point so instead we study the set of limits of all possible convergent subsequences. Formally, given a dynamical system (IR,X,ϕ) with flow ϕ:IR×X→X and a starting point x∈X, we call point y∈X an ω-limit point of the orbit through *x* if there exists a sequence ${\left(t_{n}\right)}_{n\in N}\in I\;R$ such that $\lim_{n\to \infty }t_{n}=\infty ,\;\lim_{n\to \infty }\phi \left(t_{n},x\right)=y.$ Alternatively, the ω-limit set can be defined as: $\omega_{\Phi }\left(x\right)=\cap_{t}\bar{\cup_{\tau \geq t}\phi \left(\tau ,x\right)}.$

**Definition** **4.** *We say a point x∈X is a recurrent point if there exists a sequence ${\left(t_{n}\right)}_{n\in N}\in I\;R$ such that $\lim_{n\to \infty }t_{n}=\infty ,\;\lim_{n\to \infty }\phi \left(t_{n},x\right)=x$, i.e., if $x\in \omega_{\Phi }\left(x\right)$.*

We denote the boundary of a set *X* as bd(X) and the interior of *S* as int(X). In the case of replicator dynamics where the state space *X* corresponds to a product of agent (mixed) strategies, we will denote by $\phi_{i}\left(t,x\right)$ the projection of the state on the simplex of mixed strategies of agent *i*. In our replicator system, we embed our state space with the standard topology and the Euclidean distance metric.

#### 3.3.1. Liouville’s Formula

Liouville’s formula can be applied to any system of autonomous differential equations with a continuously differentiable vector field ξ on an open domain of $S\subset {I\;R}^{k}$. The divergence of ξ at x∈S is defined as the trace of the corresponding Jacobian at *x*, i.e., $\mathrm{div}\left[\xi \left(x\right)\right]=\sum_{i=1}^{k}\frac{\partial \xi_{i}}{\partial x_{i}}\left(x\right)$. Since divergence is a continuous function, we can compute its integral over measurable sets A⊂S. Given any such set *A*, let $A\left(t\right)=\{\Phi \left(x_{0},t\right):x_{0}\in A\}$ be the image of *A* under map Φ at time *t*. A(t) is measurable and its volume is $\mathrm{vol}\left[A\left(t\right)\right]=\int_{A(t)}dx$. Liouville’s formula states that the time derivative of the volume A(t) exists and is equal to the integral of the divergence over A(t): $\frac{d}{dt}\left[A\left(t\right)\right]=\int_{A(t)}\mathrm{div}\left[\xi \left(x\right)\right]dx.$

A vector field is called divergence free if its divergence is zero everywhere. Liouville’s formula trivially implies that volume is preserved in such flows.

#### 3.3.2. Poincaré’s Recurrence Theorem

Poincaré [39] proved that in certain systems almost all trajectories return arbitrarily close to their initial position infinitely often. Specifically, if a flow preserves volume and has only bounded orbits, then for each open set there exist orbits that intersect the set infinitely often.

**Theorem** **1** ([39,40])**.** *Let (X,Σ,μ) be a finite measure space and let f:X→X be a measure-preserving transformation. Then, for any E∈Σ, the set of those points x of E such that $f^{n}\left(x\right)\notin E$ for all n>0 has zero measure. That is, almost every point of E returns to E. In fact, almost every point returns infinitely often. Namely,*

$$\mu \left(\{x\in E:\exists N\;such\;that\;f^{n}\left(x\right)\notin E\;for\;all\;n>N\}\right)=0.$$

If we take *X* to be a separable metric space as well as a finite measure space, then we can conclude that almost every point is recurrent. Cover *X* by countably many balls of radius ϵ/2, and apply the previous theorem to each ball. We conclude that almost every point of *X* returns to within an ϵ of itself. Since ϵ>0 is arbitrary, we conclude that almost every point of *X* is recurrent.

**Corollary** **1.** *Let (X,Σ,μ) be a separable metric space as well as a finite measure space and let f:X→X be a measure-preserving transformation. Then, almost every point of X is recurrent.*

#### 3.3.3. Homeomorphisms and Conjugacy of Flows

A function *f* between two topological spaces is called a *homeomorphism* if it has the following properties: *f* is a bijection, *f* is continuous, and *f* has a continuous inverse. A function *f* between two topological spaces is called a *diffeomorphism* if it has the following properties: *f* is a bijection, *f* is continuously differentiable, and *f* has a continuously differentiable inverse. Two flows $\Phi^{t}:A\to A$ and $\Psi^{t}:B\to B$ are conjugate if there exists a homeomorphism g:A→B such that for each x∈A and t∈IR: $g\left(\Phi^{t}\left(x\right)\right)=\Psi^{t}\left(g\left(x\right)\right).$ Furthermore, two flows $\Phi^{t}:A\to A$ and $\Psi^{t}:B\to B$ are *diffeomorhpic* if there exists a diffeomorphism g:A→B such that for each x∈A and t∈IR $g\left(\Phi^{t}\left(x\right)\right)=\Psi^{t}\left(g\left(x\right)\right)$. If two flows are diffeomorphic, then their vector fields are related by the derivative of the conjugacy. That is, we get precisely the same result that we would have obtained if we simply transformed the coordinates in their differential equations [41].

### 3.4. The Fundamental Theorem of Dynamical Systems

In order to characterize chain recurrent sets of replicator dynamics in games, we will exploit some tools and theorems developed about chain recurrence in more general dynamical systems. The standard formulation of the fundamental theorem of dynamical systems is built on the following set of definitions, based primarily on the work of Conley [4].

**Definition** **5.** *Let $\phi^{t}$ be a flow on a metric space (X,d). Given ϵ>0, T>0, and x,y∈X, an (ϵ,T)-chain from x to y with respect to $\phi^{t}$ and d is a pair of finite sequences $x=x_{0},x_{1},\cdots ,x_{n-1},x_{n}=y$ in X and $t_{0},\cdots ,t_{n-1}$ in [T,∞), denoted together by $\left(x_{0},\cdots ,x_{n};t_{0},\cdots ,t_{n-1}\right)$ such that*

$$d(\phi^{t_{i}}\left(x_{i}\right),x_{i+1})<\epsilon$$

*for i=0,1,2,⋯,n−1.*

**Definition** **6.** *Let $\phi^{t}$ be a flow on a metric space (X,d). The forward chain limit set of x∈X with respect to $\phi^{t}$ and d is the set*

$$\Omega^{+}\left(x\right)=\underset{\epsilon ,T>0}{⋂}\left\{y\in X\;|\;\exists \;\mathrm{an}\;\left(\epsilon ,T\right)-\mathrm{chain}\;\mathrm{from}\;x\;\mathrm{to}\;y\right\}.$$

**Definition** **7.** *Let $\phi^{t}$ be a flow on a metric space (X,d). Two points x,y∈X are chain equivalent with respect to $\phi^{t}$ and d if $y\in \Omega^{+}\left(x\right)$ and $x\in \Omega^{+}\left(y\right)$.*

**Definition** **8.** *Let $\phi^{t}$ be a flow on a metric space (X,d). A point x∈X is chain recurrent with respect to $\phi^{t}$ and d if x is chain equivalent to itself. The set of all chain recurrent points of $\phi^{t}$, denoted R(ϕ), is the chain recurrent set of $\phi^{t}$.*

One key definition is the notion of a complete Lyapunov function. The game theoretic analogue of this idea is the notion of a potential function in potential games. In a potential game, as long as we are not at an equilibrium, the potential is strictly decreasing guiding the dynamics towards the standard game theoretic solution concept, i.e., equilibria. The notion of a complete Lyapunov function switches the target solution concept from equilibria to chain recurrent points. More formally:

**Definition** **9.** *Let $\phi^{t}$ be a flow on a metric space X. A complete Lyapunov function for $\phi^{t}$ is a continuous function γ:X→IR such that*

*(i)*  *$\gamma (\phi^{t}\left(x\right))$ is a strictly decreasing function of t for all $x\in X\setminus R(\phi^{t})$,*
*(ii)*  *for all $x,y\in R(\phi^{t})$ the points x, y are chain equivalent with respect to $\phi^{t}$ if and only if γ(x)=γ(y),*
*(iii)*  *$\gamma (R\left(\phi^{t}\right))$ is nowhere dense.*

The powerful implication of the fundamental theorem of dynamical systems is that complete Lyapunov functions always exist. In game theoretic terms, every game is a “potential” game, if only we change our solution concept from equilibria to chain recurrent sets.

**Theorem** **2** ([4])**.** *Every flow on a compact metric space has a complete Lyapunov function.*

#### Alternatively, Equivalent Formulations of Chain Equivalence

For the purpose of our investigation, it will be useful to apply the following alternative definitions of chain equivalence, which are due to Hurley [42].

**Definition** **10.** *Let (X,d) be a metric space, and let f:X→X. Given ϵ>0 and x,y∈X, an ϵ-chain from x to y is a finite sequence*

$$x=x_{0},x_{1},\cdots ,x_{n-1},x_{n}=y$$

*in X such that $d(f\left(x_{i}\right),x_{i+1})<\epsilon$ for i=0,1,2,⋯,n−1.*

**Definition** **11.** *Let X be a metric space, and let f:X→X. Two points x,y∈X are called chain equivalent if for every ϵ>0 there exists an ϵ-chain from x to y and there exists an ϵ-chain from y to x.*

Next, we provide three alternative formulations of chain equivalence that are equivalent with our original definition for a flow on a compact metric space as shown in [42].

**Theorem** **3** ([42])**.** *If $\phi^{t}$ is a flow on a compact metric space (X,d) and x,y∈X, then the following statements are equivalent:*

*(i)*  The points x and y are chain equivalent with respect to $\phi^{t}$.
*(ii)*  *For every ϵ>0 and T>0, there exists an (ϵ,1)-chain*
  $$\left(x_{0},\cdots ,x_{n};t_{0},\cdots ,t_{n-1}\right)$$
  *from x to y such that*
  $$t_{0}+\cdots +t_{n-1}\geq T$$
  *and there exists an (ϵ,1)-chain*
  $$\left(y_{0},\cdots ,y_{m};s_{0},\cdots ,s_{m-1}\right)$$
  *from y to x such that*
  $$s_{0}+\cdots +s_{m-1}\geq T.$$
*(iii)*  For every ϵ>0, there exists an (ϵ,1)-chain from x to y and an (ϵ,1)-chain from y to x.
*(iv)*  The points x and y are chain equivalent with respect to $\phi^{1}$.

### 3.5. Chain Components

**Definition** **12.** *The relation ∼ defined by x∼y if and only if x is chain equivalent to y is an equivalence relation on the chain recurrent set of a flow on a metric space. An equivalence class of the chain equivalence relation for $\phi^{t}$ is a chain component of $\phi^{t}$.*

One key insight of Conley was the discovery of this formal and intuitive connection between equivalence classes and connectedness.

**Theorem** **4** ([4,38])**.** *The chain components of a flow on a compact metric space are the connected components of the chain recurrent set of the flow.*

This interplay between chain transitivity and connectedness motivates the reference to the equivalence classes of the chain equivalence relation as chain components. Furthermore, chains components are dynamically irreducible, i.e., they cannot be partitioned more finely in a way that respects the system dynamics. To make this notion precise, we need the notion of chain transitivity introduced by Conley [4].

**Definition** **13.** *Let $\phi^{t}$ be a flow on a metric space X. A set A⊂X is chain transitive with respect to $\phi^{t}$ if A is a nonempty closed invariant set with respect to $\phi^{t}$ such that for each x,y∈A, ϵ>0 and T>0 there exists an (ϵ,T)-chain from x to y.*

**Theorem** **5** ([38])**.** *Every chain component of a flow on a compact metric space is closed, connected, and invariant with respect of the flow. Moreover,*

- Every chain component of a flow on a metric space is chain transitive with respect to the flow.
- Every chain transitive set with respect to a flow on a metric space is a subset of a unique chain component of the flow.
- If A and B are chain transitive with respect to a flow on a metric space, A⊂B and C is the unique chain component containing A, then B⊂C.

## 4. Chain Recurrent Sets for Zero-Sum Games

Zero-sum games are amongst the most well studied class of games within game theory. Equilibria here are classically considered to completely “solve” the setting. This is due to the fact that the equilibrium prediction is essentially unique, Nash computation is tractable, and many natural classes of learning dynamics are known to “converge weakly” to the set of Nash equilibria.

The notion of weak convergence encodes that the time average of the dynamics converge to the equilibrium set. However, this linguistic overloading of the notion of convergence is unnatural and arguably can lead to a misleading sense of certainty about the complexity that learning dynamics may exhibit in this setting. For example, would it be meaningful to state that the moon “converges weakly” to the earth instead of stating that e.g., the moon follows a trajectory that has earth at its center?

The complexity and unpredictability of the actual behavior of dynamics becomes apparent when we characterize the set of chain recurrent points even for the simplest zero-sum games, Matching Pennies. Despite the uniqueness and symmetry of the Nash equilibrium, it is shown to not capture fully the actual dynamics. The set of chain recurrent points is the whole strategy space. This means that, in the presence of arbitrary small noise, replicator dynamics can become completely unpredictable. Even in an idealized implementation without noise, there exist absolutely no initial conditions that converge to a Nash equilibrium. This result extends to all zero-sum games that have at least one interior Nash equilibrium, i.e., a mixed strategy Nash equilibrium where both agents play all of their strategies with positive probability.

**Figure 1:**
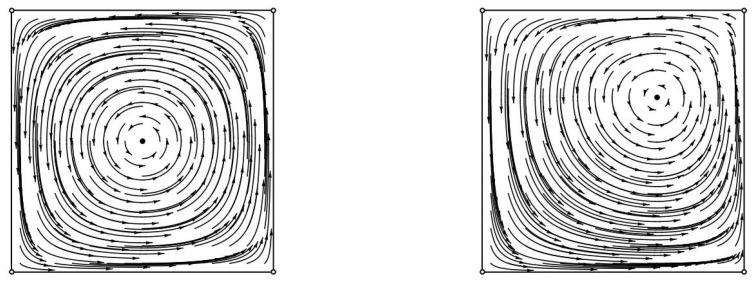
Replicator trajectories in the Matching Pennies game (**left**) and in a different zero-sum game with a fully mixed Nash equilibrium (**right**). Each point encodes the probability assigned by the agents to their first strategy. In Matching Pennies at Nash equilibrium, each agent chooses the first strategy with probability 1/2. In the second game at Nash equilibrium, each agent chooses the first strategy with probability 2/3. All interior points lie on periodic trajectories.

**Figure 2:**
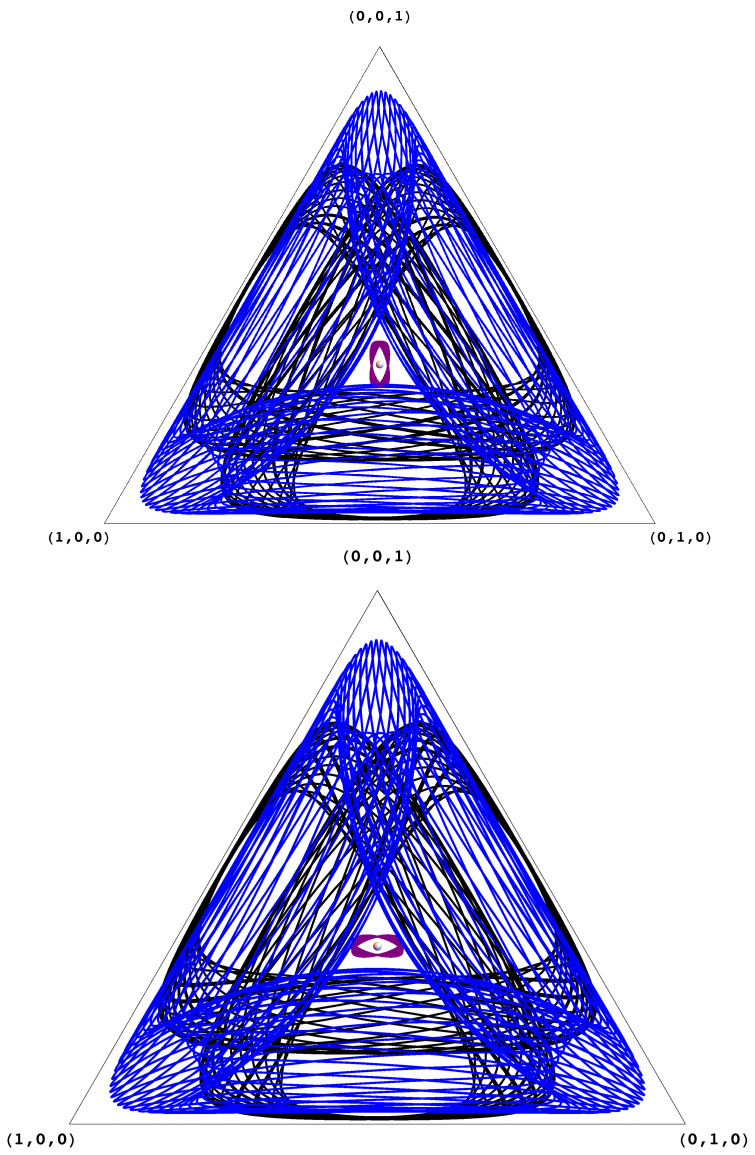
Replicator trajectories of the first (resp. second) agent in the Rock–Paper–Scissors game. Each color corresponds to a different trajectory/initial condition. The three different initial conditions are not chosen to enforce any type of symmetric behavior for the two agents. In the format $(x_{Rock},x_{Paper},x_{Scissors},y_{Rock},y_{Paper},y_{Scissors}),$ the initial conditions are the following: Black (0.5, 0.01, 0.49, 0.5, 0.25, 0.25), Blue (0.1, 0.2, 0.7, 0.8, 0.04, 0.16), and Purple (0.32, 0.3, 0.38, 0.33, 0.32, 0.35). As expected from our analysis, the trajectories for both agents are recurrent. Moreover, the purple trajectory whose initial condition is chosen to be "close" to the Nash equilibrium (in K-L divergence) stays close to it.

To argue this, we will use two lemmas. The first lemma, which follows from previous work [5,23], argues that the Kullback–Leibler divergence (KL-divergence) from the evolving state to the Nash equilibrium is constant for all trajectories. The second lemma, which is a strengthening of arguments from [23], argues that in the case of zero-sum games with fully mixed equilibria almost all states are recurrent (see also Figure 1 and Figure 2). Their proofs can be found in the Appendix B.

**Lemma** **1** ([5,23])**.** *Let φ denote the flow of replicator dynamics when applied to a zero sum game with a fully mixed Nash equilibrium $q=(q_{1},q_{2})$. Given any (interior) starting point $x\left(0\right)=(x_{1}\left(0\right),x_{2}\left(0\right))$ then the sum of the KL-divergences between each agent’s mixed Nash equilibrium $q_{i}$ and his evolving strategy $x_{i}\left(t\right)$ is time invariant. Equivalently, $D_{\mathrm{KL}}\left(q_{1}∥x_{1}\left(t\right)\right)+D_{\mathrm{KL}}\left(q_{2}∥x_{2}\left(t\right)\right)=D_{\mathrm{KL}}\left(q_{1}∥x_{1}\left(0\right)\right)+D_{\mathrm{KL}}\left(q_{2}∥x_{2}\left(0\right)\right)$ for all t.*

**Lemma** **2.** *Let φ denote the flow of replicator dynamics when applied to a zero sum game with a fully mixed Nash equilibrium. Then, all but a zero-measure set of its initial conditions are recurrent.*

The main theorem of this section is the following:

**Theorem** **6.** *Let φ denote the flow of replicator dynamics when applied to a zero sum game with a fully mixed Nash equilibrium. Then, the set of chain recurrent points is the whole state space. Its unique chain component is the whole state space.*

**Proof.**  By Lemma 2, all but a zero measure set of initial conditions are recurrent (and hence trivially chain-recurrent). By Theorem 5, all chain components and thus the chain recurrent set are closed. By taking closure over the set of all recurrent points, we have that all points in the state space must be chain recurrent. By Theorem 4, since the chain components of a flow on a compact metric space are the connected components of its chain recurrent set, the whole state space is the unique chain component. ☐

It is easy to generalize the above proofs in the case where the two-agent game is a weighted zero-sum game, i.e., in the case where $A+cB^{T}=0$ for some c>0, where A,B are the payoff matrices of the two agents. In this case, the weighted sum of the KL-divergences remains constant and the rest of the proof holds as long as the game has a fully mixed Nash equilibrium. In fact, the proof generalizes even in the case where we have a network polymatrix game, where each agent corresponds to a node on a undirected graph and each edge correspond to a zero-sum game. In this case, the sum of the KL divergences of all agents remains constant and the rest of the proof holds as is. See also [18,23].

## 5. Chain Recurrent Sets for Weighted Potential Games

We show that under replicator dynamics the chain recurrent sets for any (weighted) potential games coincides with the set of system equilibria. The set of chain recurrent points of gradient-like systems can be rather complicated and Conley [4] constructs a specific example of a gradient-like system with a set of equilibria of zero measure where the set of chain recurrent points is the whole state space. Nevertheless, we establish that such contrived examples do not arise in (weighted) potential games. For the proof of this characterization, we will apply the following theorem due to Hurley that we have already discussed in the preliminaries:

**Theorem** **7** ([42])**.** *The chain recurrent set of a continuous (semi)flow on an arbitrary metric space is the same as the chain recurrent set of its time-one map.*

**Theorem** **8.** *Let φ denote the flow of replicator dynamics when applied to a weighted potential game. Then, the set of chain recurrent points coincides with its set of equilibria. The chain components of the flow are exactly the connected components of the set of equilibria.*

**Proof.**  Replicator dynamics defines a gradient-like system, where the (expected) value of the potential function always increases unless we are at a fixed point. Specifically, it is well known that in any potential game the utility of any agent at a state *s*, $u_{i}\left(s\right)$ can be expressed as a summation of the potential Φ and a dummy term $D_{i}\left(s_{-i}\right)$ that depends on the strategies of all of the other n−1 agents other than *i*. Similarly, by the definition of the weighted potential game for any possible deviation of agent *i* from strategy $s_{i}$ to $s_{i}^{\prime }$: $\frac{\Phi \left(s_{i}^{\prime },s_{-i}\right)-\Phi \left(s_{i},s_{-i}\right)}{w_{i}}=u_{i}\left(s_{i}^{\prime },s_{-i}\right)-u_{i}\left(s_{i},s_{-i}\right)$ and hence for each $s_{-i}=\times_{j\neq i}s_{j}$ and any two possible strategies $s_{i},s_{i}^{\prime }$ of agent *i*, we have that $u_{i}\left(s_{i}^{\prime },s_{-i}\right)-\frac{\Phi (s_{i}^{\prime },s_{-i})}{w_{i}}=u_{i}\left(s_{i},s_{-i}\right)-\frac{\Phi (s_{i},s_{-i})}{w_{i}}$. Hence, these differences are independent of the choice of strategy of agent *i* and can be expressed as $D_{i}\left(s_{-i}\right)$, a function of the choices of all other agents. We can now express $u_{i}\left(s\right)=\frac{\Phi (s)}{w_{i}}+D_{i}\left(s_{-i}\right)$ and similarly $u_{i}\left(x\right)=\frac{\Phi (x)}{w_{i}}+D_{i}\left(x_{-i}\right)$ for mixed strategy profiles. Furthermore, we have that since $\Phi \left(x\right)=\sum_{s_{i}\in S_{i}}x_{s_{i}}\Phi \left(s_{i},x_{-i}\right)$, we have that for each $s_{i}\in S_{i}$$\frac{\partial \Phi (x)}{\partial x_{s_{i}}}=\Phi \left(s_{i},x_{-i}\right)$. Therefore, we have that:

(1) $$\begin{array}{ccc} \frac{\partial \Phi (x)}{\partial t} & = & \sum_{i}\sum_{s_{i}\in S_{i}}\frac{\partial \Phi (x)}{\partial x_{s_{i}}}{\dot{x}}_{s_{i}}=\sum_{i}\sum_{s_{i}\in S_{i}}\Phi \left(s_{i},x_{-i}\right){\dot{x}}_{s_{i}}\\ & = & \sum_{i}\sum_{s_{i}\in S_{i}}\Phi \left(s_{i},x_{-i}\right)x_{s_{i}}\left[u_{i}\left(s_{i},x_{-i}\right)-u_{i}\left(x\right)\right]\\ & = & \sum_{i}\frac{1}{w_{i}}\sum_{s_{i}\in S_{i}}\Phi \left(s_{i},x_{-i}\right)x_{s_{i}}\left[\Phi \left(s_{i},x_{-i}\right)-\Phi \left(x\right)\right]\\ & = & \frac{1}{2}\sum_{i,s_{i},s_{i}^{\prime }\in S_{i}}\frac{1}{w_{i}}x_{s_{i}}x_{s_{i}^{\prime }}{\left[\Phi \left(s_{i},x_{-i}\right)-\Phi \left(s_{i}^{\prime },x_{-i}\right)\right]}^{2}\\ & = & \frac{1}{2n}\;\sum_{i,s_{i},s_{i}^{\prime }\in S_{i}}\;x_{s_{i}}x_{s_{i}^{\prime }}\sum_{i,s_{i},s_{i}^{\prime }\in S_{i}}\;\frac{1}{w_{i}}x_{s_{i}}x_{s_{i}^{\prime }}{\left[\Phi \left(s_{i},x_{-i}\right)-\Phi \left(s_{i}^{\prime },x_{-i}\right)\right]}^{2}\\ & \geq & \frac{1}{2n}{\left[\sum_{i,s_{i},s_{i}^{\prime }\in S_{i}}\frac{1}{\sqrt{w_{i}}}x_{s_{i}}x_{s_{i}^{\prime }}\left|\Phi \left(s_{i},x_{-i}\right)-\Phi \left(s_{i}^{\prime },x_{-i}\right)\right|\right]}^{2}\\ & = & \frac{1}{2n}{\left[\sum_{i,s_{i},s_{i}^{\prime }\in S_{i}}\sqrt{w_{i}}x_{s_{i}}x_{s_{i}^{\prime }}\left|u_{i}\left(s_{i},x_{-i}\right)-u_{i}\left(s_{i}^{\prime },x_{-i}\right)\right|\right]}^{2}\\ & \geq & \frac{1}{2n}{\left[\sum_{i,s_{i}\in S_{i}}\sqrt{w_{i}}x_{s_{i}}|\sum_{s_{i}^{\prime }\in S_{i}}x_{s_{i}^{\prime }}\left(u_{i}\left(s_{i},x_{-i}\right)-u_{i}\left(s_{i}^{\prime },x_{-i}\right)\right)|\right]}^{2}\\ & = & \frac{1}{2n}{\left[\sum_{i,s_{i}\in S_{i}}\sqrt{w_{i}}x_{s_{i}}|u_{i}\left(s_{i},x_{-i}\right)-u_{i}\left(x\right)|\right]}^{2}\geq \frac{1}{2n}\min_{i}w_{i}{∥\xi ∥}_{1}^{2}, \end{array}$$

where ξ expresses the replicator vector field. This suffices to argue convergence to equilibria for weighted potential games (expanding an analogous construction in [8] for congestion/potential games). We will furthermore use this argument to establish that the set of potential values attained at equilibrium points, i.e., V={Φ(x),xisanequilibrium} is of measure zero.

We will argue this by showing that V can be expressed as the finite union of zero measure sets. By the above derivation, we have that the potential is strictly decreasing unless we are at a system equilibrium. Equation (1) implies that in the places where the potential does not increase, i.e., at equilibria, we have that for all agents *i* if $x_{s_{i}},x_{s_{i}^{\prime }}>0$, then $\Phi \left(s_{i},x_{-i}\right)-\Phi \left(s_{i}^{\prime },x_{-i}\right)=0$. However, this immediately implies that $\frac{\partial \Phi (x)}{\partial x_{s_{i}}}-\frac{\partial \Phi (x)}{\partial x_{s_{i}^{\prime }}}=0$. Any equilibrium either corresponds to a pure state, in which cases the union of their potential values is trivially of zero measure or its corresponds to a mixed state where one or more agents is randomizing. In order to account for the possibility of continuums of equilibria, we will use Sard’s theorem that implies that the set of critical values (that is, the image of the set of critical points of a smooth function from one Euclidean space to another) is a null set, i.e., it has Lebesgue measure 0. We define an arbitrary fixed ordering over the strategy set of each agent. Given any mixed system equilibrium *x*, the expected value of the potential Φ(x) can be written as a multi-variate polynomial over all strategy variables $x_{s_{i}}$ played with strictly positive probability. Since the $x_{s_{i}}$’s represent probabilities, we can replace the lexicographically smaller variable $x_{s_{i}}$ as one minus the summation of all other variables in the support of the current mixed strategy of agent *i*. Now, however, all partial derivatives of this polynomial at the equilibrium are equal to zero. Hence, each equilibrium can be expressed as a critical point of a smooth function from some space ${I\;R}^{k}$ to IR and hence its image (i.e., its set of potential values) is a zero measure subset of IR. It is clear that the set of polynomials needed to capture all equilibria depends only on the choice of strategies for each agent that are played with positive probability and hence although they are exponential many they are finite. Putting everything together the set of potential values attained at equilibria is of zero measure.

Naturally, however, the complement of equilibrium values, which we denote C, is dense in the set $\left[\min_{s}\Phi \left(s\right),\max_{s}\Phi \left(s\right)\right]$. Indeed, if C is not dense in $[\min_{s}\Phi \left(s\right),\max_{s}\Phi \left(s\right)],$ then there exists a point $y\in [\min_{s}\Phi \left(s\right),\max_{s}\Phi \left(s\right)]$ such that y∉C and at the same time *y* is not an accumulation point of C. This implies that there exists a neighborhood of *y* that contains no points of C. We reach contradiction since $[\min_{s}\Phi \left(s\right),\max_{s}\Phi \left(s\right)]\setminus C$ is of zero measure.

Next, we will use the fact that the complement of equilibrium values of the potential is dense in the set $[\min_{s}\Phi \left(s\right)$, $\max_{s}\Phi \left(s\right)]$ to establish that the chain recurrent points of the time one map $\phi^{1}$ of the flow coincide with the set of equilibria. As we stated above, Hurley [42] has shown that the chain recurrent points of the flow coincide with those of its time one map and hence the theorem follows.

We have that $\Phi (\phi^{1}\left(x\right))\geq \Phi \left(x\right)$ for all *x*, with equality if and only if we are at equilibrium. Suppose that we choose a regular value *r* of the potential, i.e., a value that does not correspond to a fixed point. Let us consider the sets $K_{r}=\Phi^{-1}\left(\left[r,\infty \right)\right)$, and $U_{r}=\Phi^{-1}\left(\left(r,\infty \right)\right)$. Note that $K_{r}$ is closed while $U_{r}$ is open (in the topology defined by the set of strategy profiles) and contained in $K_{r}$. If Φ(p)=r, then $\Phi (\phi^{1}\left(p\right))>\Phi \left(p\right)=r$. This means that $\phi^{1}\left(K_{r}\right)\subset U_{r}$. However, since $\phi^{1}$, the time one map of the flow is a homeomorphism, the fact that $K_{r}$ is closed yields that $\bar{\phi^{1}\left(U_{r}\right)}\subset \bar{\phi^{1}\left(K_{r}\right)}=\phi^{1}\left(K_{r}\right)\subset U_{r}$.

Any chain recurrent point whose forward orbits meets $U_{r}$ is furthermore contained in $\bar{\phi^{1}\left(U_{r}\right)}$ [4,42]. Let *q* be a non-equilibrium point; then, we have that $\Phi (\phi^{1}\left(q\right))>\Phi \left(q\right)$. However, due to the fact that the images of the potential values of non-equilibrium points are dense in $\left(\min_{s}\Phi \left(s\right),\max_{s}\Phi \left(s\right)\right)$, we can choose such a value *r* such that $\Phi (\phi^{1}\left(q\right))>r>\Phi \left(q\right)$. Then $\phi^{1}\left(q\right)\in U_{r}$ but $q\notin U_{r}$ and thus $q\notin \bar{\phi^{1}\left(U_{r}\right)}$. Therefore, *q* cannot be a chain recurrent point of $\phi^{1}$. As such, it cannot be a chain recurrent point for the replicator flow as well. The theorem follows immediately since all equilibria are trivially chain recurrent points.

Finally, by Theorem 4, since the chain components of a flow on a compact metric space are the connected components of its chain recurrent set and for weighted potential games the chain recurrent set is the set of equilibria, the chain components of the flow are exactly the connected components of the set of equilibria. ☐

## 6. Discussion and Open Questions

Nash equilibrium is the standard solution concept of game theory. It provides a behavioral model that is applicable to any game. Nevertheless, Nash equilibria are not without criticism. They are hard to compute in practice even in simple games [43], they are rarely chosen when they are not Pareto efficient [44], and they rarely agree with the behavior of learning/evolutionary dynamics [45].

In this paper, we shift the focus away from Nash equilibria and on to the dynamics themselves. However, instead of focusing on the fixed points of the dynamics (which would lead back to Nash equilibria and related notions), we focused on their chain recurrent points. This is a natural relaxation of periodic points that is justified as a solution concept via Conley’s fundamental theorem of dynamical systems [4].

We analyzed two simple and evocative examples, namely zero-sum games with fully mixed equilibria and weighted potential games under replicator dynamics, the most well known evolutionary dynamics. Interestingly, even in simple 2×2 games, the set of chain recurrence points gives radically different predictions than Nash equilibria. In games with strongly misaligned incentives (e.g., Matching Pennies), this set tends to be large implying unpredictability of the dynamics, whereas in games with strongly aligned incentives it tends to be small implying that learning/adaptation is fundamentally easier in collaborative environments. Naturally, there is much that needs to be done, and below we sample a few research goals that are immediate, important, and each open ended in its own way.

**Figure 3:**
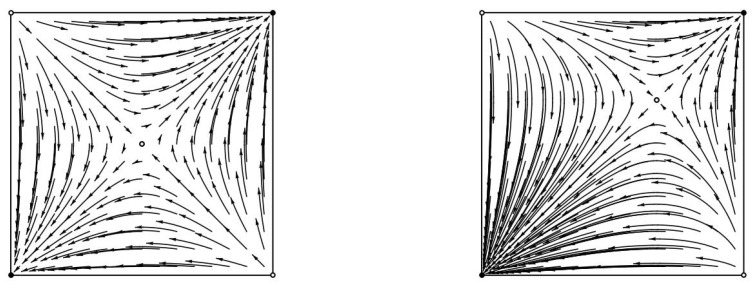
Replicator trajectories in a doubly symmetric partnership, coordination game (**left**) and in a different coordination game with a fully mixed Nash equilibrium (**right**). Each point encodes the probability assigned by the agents to their first strategy. In the first game at Nash equilibrium, each agent chooses the first strategy with probability 1/2. In the second game at Nash equilibrium, each agent chooses the first strategy with probability 2/3. All trajectories converge to equilibria.

- **The structure of the Chain Recurrent Set (CRS) and the Chain Components (CCs).** A game may have many chain components (for example, the coordination games in Figure 3 has five). It is not hard to see that the chain components can be arranged as vertices of a directed acyclic graph, where directed edges signify possible transitions after an infinitesimal jump; for the coordination games in Figure 3, this directed acyclic graph (DAG) has two sinks (the pure Nash equilibria), two sources (the other two pure profiles), and a node of degree 4 (the mixed Nash equilibrium). Identifying this DAG is tantamount to analyzing the game, the generalization of finding its Nash equilibria. Understanding this fundamental structure in games of interest is an important challenge.
- **Price of Anarchy through Chain Recurrence.** We can define a natural distribution over the sink chain components (CCs) of a game, namely, assign to each sink CC the probability that a trajectory started at a (say, uniformly) random point of the state space will end up, perhaps after infinitesimal jumps, at the CC. This distribution, together with the CC’s expected utility, yield a new and productive definition of the average price of anarchy in a game, as well as a methodology for calculating it (see, for example, [46]).
- **Inside a Chain Component.** Equilibria and limit cycles are the simplest forms of a chain components, in the sense that no “jumps” are necessary for going from one state in the component to another. In Matching Pennies, in contrast, $O(\frac{1}{\epsilon }),$ many ϵ-jumps are needed to reach the Nash equilibrium, starting from a pure strategy profile. What is the possible range of this form of complexity of a CC?
- **Complexity.** There are several intriguing complexity questions posed by this concept. What is the complexity of determining, given a game and two strategy profiles, whether they belong to the same chain component (CC)? What is the complexity of finding a point in a sink CC? What is the speed of convergence to a CC?
- **Multiplicative Weights Update and Discrete-time Dynamics through Chain Components.** We have explicitly discussed the connection between replicator dynamics and MWU. At the same time, it has recently been shown that for large step-sizes ϵ MWU(ϵ) can behave chaotically even in two agent two strategy coordination/potential games [17]. Is it possible to understand this chaotic behavior through the lens of CCs? Moreover, can we understand and predict the bifurcation from the convergent behavior of replicator dynamics (i.e., MWU(ϵ) with ϵ→0) to the chaotic behavior of MWU(ϵ) with large step-size ϵ?
- **Information Geometry, Social Welfare and the Fundamental Theorem of Dynamical Systems.** In the case of zero-sum games, each point of a given replicator trajectory lies at a fixed KL-divergence from the Nash equilibrium. Similar KL-divergence invariant properties also apply in the case of (network) coordination games [46]. It is not known whether any (information theoretic) invariant properties applies, e.g., to a general two person game for replicator dynamics. The fundamental theorem of dynamical systems shows the existence of a complete Lyapunov function that is invariant on the chain recurrence set (and hence on each chain component) but strictly decreases outside this set. Can we express this function for the replicator flow in a general two-person game as a combination of information theoretic properties (e.g., KL-divergences) and game theoretic properties (e.g., the sum of utilities of all agents)?

## Appendix A Information Theory

Entropy captures the expected information value from a measurement of a random variable. The entropy *H* of a discrete random variable *X* with possible values {1,⋯,n} and probability mass function p(X) is defined as $H\left(X\right)=-\sum_{i=1}^{n}p(i)\ln p(i).$

Given two probability distributions *p* and *q* of a discrete random variable their *KL-divergence* (relative entropy) is defined as $D_{\mathrm{KL}}\left(p∥q\right)=\sum_{i}\ln\left(\frac{p(i)}{q(i)}\right)p\left(i\right).$ It is the average of the logarithmic difference between the probabilities *p* and *q*, where the average is taken using the probabilities *p*. The KL-divergence is only defined if q(i)=0 implies p(i)=0 for all *i* (The quantity 0ln0 is interpreted as zero because $\lim_{x\to 0}x\ln\left(x\right)=0$.) KL-divergence is a “pseudo-metric” in the sense that it is always non-negative and is equal to zero if and only if the two distributions are equal (almost everywhere). Other useful properties of the KL-divergence is that it is additive for independent distributions and that it is jointly convex in both of its arguments; that is, if $\left(p_{1},q_{1}\right)$ and $\left(p_{2},q_{2}\right)$ are two pairs of distributions then for any 0≤λ≤1: $D_{\mathrm{KL}}\left(\lambda p_{1}+\left(1-\lambda \right)p_{2}∥\lambda q_{1}+\left(1-\lambda \right)q_{2}\right)\leq \lambda D_{\mathrm{KL}}\left(p_{1}∥q_{1}\right)+\left(1-\lambda \right)D_{\mathrm{KL}}\left(p_{2}∥q_{2}\right)$. For more details and proofs of these basic facts, the reader should refer to the classic text by Cover and Thomas [47].

## Appendix B Missing Proofs of Section 4

**Lemma** **A1.**
*Let φ denote the flow of replicator dynamics when applied to a zero sum game with a fully mixed Nash equilibrium $q=(q_{1},q_{2})$. Given any (interior) starting point $x\left(0\right)=(x_{1}\left(0\right),x_{2}\left(0\right)),$ then the sum of the KL-divergences between each agent’s mixed Nash equilibrium $q_{i}$ and his evolving strategy $x_{i}\left(t\right)$ is time invariant. Equivalently, $D_{\mathrm{KL}}\left(q_{1}∥x_{1}\left(t\right)\right)+D_{\mathrm{KL}}\left(q_{2}∥x_{2}\left(t\right)\right)=D_{\mathrm{KL}}\left(q_{1}∥x_{1}\left(0\right)\right)+D_{\mathrm{KL}}\left(q_{2}∥x_{2}\left(0\right)\right)$ for all t.*

**Proof.** It suffices to show that the time derivative of $D_{\mathrm{KL}}\left(q_{1}∥x_{1}\left(t\right)\right)+D_{\mathrm{KL}}\left(q_{2}∥x_{2}\left(t\right)\right)$ is everywhere equal to zero. By properties of the KL-divergence, it suffices to show that the time derivative of the quantity $\sum_{i}H\left(q_{i},\phi_{i}\left(t,x_{0}\right)\right)=-\sum_{i}\sum_{s_{i}\in S_{i}}q_{s_{i}}\cdot \ln\left(x_{s_{i}}\right)$ is everywhere zero, where i∈{1,2} and $S_{i}$ the available strategies of agent *i*. We denote by $A^{1,2}$ (resp. $A^{2,1}$) the payoff matrix of agent 1 (resp. agent 2). Since this is a zero-sum game: $A^{1,2}+{\left(A^{2,1}\right)}^{T}=0$ where ${\left(A^{2,1}\right)}^{T}$ the transpose of $A^{2,1}$.

$$\begin{array}{c} \sum_{i}\sum_{s_{i}\in S_{i}}q_{s_{i}}\frac{d\ln(x_{s_{i}})}{dt}=\sum_{i}\sum_{s_{i}\in S_{i}}q_{s_{i}}\frac{{\dot{x}}_{s_{i}}}{x_{s_{i}}}\\ \;=\sum_{i}\left(\sum_{s_{i}\in S_{i}}q_{s_{i}}u_{i}\left(s_{i},x_{-i}\right)-\sum_{s_{i}\in S_{i}}x_{s_{i}}u_{i}\left(s_{i},x_{-i}\right)\right)\\ \;=\left(q_{1}^{T}A^{1,2}x_{2}-x_{1}^{T}A^{1,2}x_{2}\right)+\left(q_{2}^{T}A^{2,1}x_{1}-x_{2}^{T}A^{2,1}x_{1}\right)\\ \;=\left(q_{1}^{T}-x_{1}^{T}\right)A^{1,2}\left(x_{2}-q_{2}\right)+\left(q_{2}^{T}-x_{2}^{T}\right)A^{2,1}\left(x_{1}-q_{1}\right)\\ \;=-\left(q_{1}^{T}-x_{1}^{T}\right)\left[A^{1,2}+{\left(A^{2,1}\right)}^{T}\right]\left(q_{2}-x_{2}\right)=0. \end{array}$$

☐

In the case of zero-sum games with no fully mixed equilibria, the K-L divergence to the barycenter equilibrium (the barycenter of the convex Nash equilibrium polytope) can be shown to strictly decrease for all interior points, which implies convergence to the subspace (subsimplex) that corresponds to the strategies played at that equilibrium [18,23]. For example, strategies survive in the long run if and only if they are chosen with positive probability in some equilibrium. Within this subspace, the dynamics are recurrent, however, in this paper we will not expand on the details of this case.

**Lemma** **A2.**
*Let φ denote the flow of replicator dynamics when applied to a zero sum game with a fully mixed Nash equilibrium. Then, all but a zero-measure set of its initial conditions are recurrent.*

**Proof.** We will show that for any n∈N all but a zero-measure set of the points in the invariant set $S_{n}=\left\{\left(x_{1},x_{2}\right)\in \Delta \left(S_{1}\right)\times \Delta \left(S_{2}\right):D_{\mathrm{KL}}\left(q_{1}∥x_{1}\right)+D_{\mathrm{KL}}\left(q_{2}∥x_{2}\right)\leq n\right\}$ are recurrent. By taking countable union over n∈N, we cover the whole interior of the statespace and the theorem follows. Let *q* be the fully mixed (i.e., interior) fixed point of the flow. By Lemma 1, the K-L divergence from the state of the system to the Nash equilibrium *q* is a constant of the motion. This constant of the motion implies that starting off any interior point, the system trajectory will always remain bounded away from the boundary. Furthermore, the system defined by applying replicator on the interior of state space can be transformed to a divergence free system on ${\left(-\infty ,+\infty \right)}^{\sum_{i}\left(|S_{i}|-1\right)}$ via the following invertible smooth map $z_{iR}=\ln\left(x_{iR}/x_{i0}\right)$, where $0\in S_{i}$ a specific (but arbitrarily chosen) strategy of agent *i*. This map $g:\times_{i}\mathrm{int}\left(\Delta \left(S_{i}\right)\right)\to {I\;R}^{\sum_{i}\left(|S_{i}|-1\right)}$ is clearly a diffeomorphism. (The inverse transformation is $g^{-1}\left(\vec{z}\right)=\left\{\begin{array}{cc} x_{i0}=\frac{1}{1+\sum_{R}e^{z_{iR}}} & \forall i,\\ x_{iR}=\frac{e^{z_{iR}}}{1+\sum_{R}e^{z_{iR}}} & \forall i\forall R\neq 0 \end{array}\right.$). However,

$$\begin{array}{ccc} \frac{d\left(\frac{x_{iR}}{x_{i0}}\right)}{dt} & = & \frac{{\dot{x}}_{iR}x_{i0}-{\dot{x}}_{i0}x_{iR}}{x_{i0}^{2}}=\frac{x_{iR}}{x_{i0}}\left(u_{i}\left(R,x_{-i}\right)-u_{i}\left(0,x_{-i}\right)\right). \end{array}$$

This implies that $\dot{z_{iR}}=\frac{d\left(\ln\frac{x_{iR}}{x_{i0}}\right)}{dt}=u_{i}\left(R,x_{-i}\right)-u_{i}\left(0,x_{-i}\right)$ where $u_{i}\left(R,x_{-i}\right),u_{i}\left(0,x_{-i}\right)$ depend only on the mixed strategies of the rest of the agents (i.e., other than *i*). As a result, the flow (II) that arises from our system via the change of variables $z_{iR}=\ln\left(x_{iR}/x_{i0}\right)$ is separable in the sense that the evolution of $z_{iR}$ depends only on the state variables of the other agents. The diagonal of the Jacobian of system (II) is zero and consequently the divergence (which corresponds to the trace of the Jacobian) is zero as well. Liouville’s theorem states that such flows are volume preserving. On the other hand, this transformation blows up the volume near the boundary to infinity and as a result does not allow for an immediate application of Poincaré’s recurrence theorem. We focus on the restriction of system (II) over the closed and bounded set $g(S_{n})$. $g(S_{n})$ is closed since $S_{n}$ is closed and *g* is a diffeomorphism and it is bounded since *n* is finite. The constant of the motion for replicator dynamics implies that replicator maps $S_{n}$ to itself. Due to the diffeomorphism *g*, the same applies for flow (II) and $g(S_{n})$. The restriction of system (II) on $g(S_{n})$ is a conservative system, since its vector field defines a measure (here Euclidean volume) preserving flow and has only bounded orbits. As a result, we can now apply Poincaré’s theorem to derive that all but a zero measure set of points of $g(S_{n})$ are recurrent. Since the diffeomorphism maps zero measure sets to zero measure sets, all but a zero-measure set of points of $S_{n}$ are recurrent and the theorem follows. ☐
